# Change in clinical outcomes during the transition of adjuvant chemotherapy for stage III colorectal cancer

**DOI:** 10.1371/journal.pone.0176745

**Published:** 2017-05-31

**Authors:** Hiroki Osumi, Eiji Shinozaki, Mitsukuni Suenaga, Takeru Wakatsuki, Izuma Nakayama, Tomohiro Matsushima, Mariko Ogura, Takashi Ichimura, Daisuke Takahari, Keisho Chin, Toshiya Nagasaki, Tsuyoshi Konishi, Takashi Akiyoshi, Yoshiya Fujimoto, Satoshi Nagayama, Yosuke Fukunaga, Masashi Ueno, Kensei Yamaguchi

**Affiliations:** 1 Departments of Gastroenterology, Cancer Institute Hospital, Japanese Foundation for Cancer Research, Tokyo, Japan; 2 Departments of Surgery, Cancer Institute Hospital, Japanese Foundation for Cancer Research, Tokyo, Japan; Baylor University Medical Center, UNITED STATES

## Abstract

**Background:**

There are robust data supporting the contribution of oxaliplatin (L-OHP) regarding clinical outcomes for colorectal cancer (CRC) in an adjuvant setting in European and US trials; however, there is no Japanese clinical evidence although L-OHP has been approved since 2009. We examined the transition of adjuvant chemotherapy for stage III colorectal cancer in our institute.

**Methods:**

A total of 642 patients with histopathologically confirmed stage III CRC underwent curative surgery from 2005 to 2010. We examined disease free survival (DFS), overall survival (OS) and prognostic factors for stage III CRC patients who underwent adjuvant chemotherapy.

**Results:**

A total of 509 patients received adjuvant chemotherapy. 3-year DFS and 5-year OS rates were 74.5% and 87.5%, respectively. The frequency of inclusion of L-OHP as adjuvant chemotherapy was increased after 2008. A total of 189 patients received adjuvant chemotherapy from 2005 to 2007 increasing to 320 patients from 2008 to 2010; the 5-year OS rates were 82.4% and 91.5%, respectively, and the 3-year DFS rates were 69.2% and 76.6%, respectively (OS, *P* = 0.007; DFS, *P* = 0.023). In univariate analysis, adjuvant chemotherapy including L-OHP was no significant deference compared to FU monotherapy. (OS: HR 0.88, 95%CI 0.4–1.91, p = 0.75, DFS: HR 0.78, 95%CI 0.21–2.3, p = 0.29). In multivariate analysis, the OS was predicted by means of N stage (HR = 2; 95%CI, 1.1–3.8; *P* = 0.02) and pathology (HR = 0.28; 95%CI, 0.13–0.59; *P* = 0.0008). The DFS was predicted by means of N stage (HR = 2.67; 95%CI, 1.82–3.9; *P* < 0.05), T stage (HR = 1.61; 95%CI, 1.1–2.3; *P* = 0.01) pathology (HR = 0.47; 95%CI, 0.29–0.75; *P* < 0.05) and venous invasion (HR = 2.06; 95%CI, 1.12–3.77; *P* = 0.01).

**Conclusions:**

Clinical outcomes of stage III CRC patients receiving adjuvant chemotherapy improved. The frequency of L-OHP usage was increasing annually, however it was no influence for clinical outcomes in this study. It will be necessary to reevaluate additional effect of L-OHP with more patients.

## Introduction

Approximately 50,000 people per year die from colorectal cancer. It is the second-leading cause of cancer deaths. Furthermore, both the mortality rate and the prevalence of colorectal cancer have increased in Japan over recent decades. [1][2] Worldwide, it is the third most prevalent cancer, and the fourth-leading cause of cancer death. [3].

Stage I and II colorectal cancer (CRC) have good survival rates after treatment involving surgery or endoscopic therapy alone; however, the 5-year survival rate of patients with stage III CRC, especially stage IIIb (T any, N2: using the Japanese classification of colorectal cancer) is worse (56.0%), because the recurrence rate is high (stage III CRC, 30.8%).[4] The use of adjuvant chemotherapy could be contributing to a reduction in mortality and recurrence.

Patients treated with 5-fluoropyrimidine (FU)/leucovorin (LV) as adjuvant chemotherapy after surgery have been reported to exhibit prolonged survival relative to patients treated with surgery alone. [5–11] Subsequently treatments have been developed involving oral adjuvant chemotherapy, for example uracil/tegafur (UFT)/LV[12], capecitabine [13] and S-1[14] have improved clinical outcomes. A previous meta-analysis that assessed the survival and disease-free survival benefits of patients treated with oral fluoropyrimidines for 1 year after surgical resection of the primary colorectal tumor reported that the overall HR was 0.89 for survival (95%CI, 0.80–0.99; *P* = 0.04), and 0.85 for disease-free survival (95%CI, 0.77–0.93; *P* = 0.001).[15]

Several studies have demonstrated the contribution to clinical outcomes of oxaliplatin (L-OHP) in an adjuvant setting for stage III colon cancer in the United States and Europe. In the MOSAIC[16], NSABP-C07 [17] and NO16968[18] trials (fluorouracil and LV [FL] alone or with L-OHP), the 3-year disease free survival (DFS) rates were 78.2%, 71.8% and 70%, respectively (MOSAIC trial, HR = 0.77 [p = 0.002]; NSABP-C07 trial, HR = 0.80 [p = 0.0034]; NO16968 trial, HR = 0.80 [p = 0.0045]). Furthermore, pooled analysis of the additive effect of L-OHP regarding clinical outcomes (overall survival [OS], DFS and time to recurrence) involving four clinical trials (NSABP C-05, C-06, C-07 and C-08) revealed that the effectiveness of L-OHP remained significant concerning DFS and OS in patients with stage III CRC.[19] In contrast, no data are available and there is controversy in Japan as to whether adding L-OHP as adjuvant chemotherapy for all patients with stage III CRC yields improved clinical outcomes, although, L-OHP has been approved for use since 2009. Furthermore, the ACTS-CC trial [14] involving S-1 or UFT/LV reported that the 3-year DFS rate using S-1 monotherapy for stage III colon cancer was 75.5% and the JCOG0205 trial [13] involving 5-FU/LV vs UFT/LV reported that the 3-year DFS rate using UFT/LV was 72.5%; this seems to be comparable to the data reported regarding the addition of L-OHP to chemotherapy regimens in Europe and US. Thus, it is considered that additional effects of L-OHP for stage III CRC may be small and differ between Japan and the US and Europe. However, there is a few long-term data to evaluate clinical outcomes and prognostic factors of stage III colon cancer patients, especially after the approval of L-OHP as adjuvant chemotherapy. The aim of the present study was to evaluate the transition of adjuvant chemotherapy and clinical outcomes for stage III CRC in our institute before and after the inclusion of L-OHP approved by health insurance.

## Patients and methods

### Patients

This study was performed in accordance with the Declaration of Helsinki. The Cancer Institute Hospital of Japanese Foundation for Cancer Research, Institutional Review Board approved the study (registry number: 1634). The study was retrospective and included 642 consecutive patients who had undergone curative surgical resection for histologically proven tumor-node-metastasis stage III CRC from January 2005 to December 2010 at the Cancer Institute Hospital. We included stage III CRC patients performing adjuvant chemotherapy. The choice of chemotherapeutic regimen depended on the date at which treatment started. According to the transition of adjuvant chemotherapy regimens, we divided the patients into two groups: those who received treatment before the use of L-OHP and those who received treatment when the frequency of use of L-OHP had been increasing.

### Treatment

The FU regimen was as follows: FU/LV (n = 3), patients received six cycles of rapid intravenous infusion of LV at a dose of 20 mg/m^2^ followed immediately by an intravenous bolus of FL at a dose of 425 mg/m^2^, on days 1 through 5 every 28 days. Six courses were administered. The UFT/LV regimen (n = 229) was as follows: patients received UFT (300 mg with a body surface area [BSA] <1.17 m^2^, 400 mg with a BSA of 1.17–1.49 m^2^, 500 mg with a BSA of 1.50–1.83 m^2^ and 600 mg with a BSA >1.83 m^2^), and LV (75 mg/body) orally in three divided doses (every 8 h) at >1 h before or after meals for 28 consecutive days, followed by a 7-day rest. Five courses were administered. The XELODA regimen (n = 173) was as follows: patients received 24 weeks of treatment with either eight cycles of oral capecitabine at a dose of 1250 mg/m^2^ of BSA, twice daily on days 1 through 14 every 21 days. The TS-1 regimen (n = 2) was as follows: patients received S-1 orally at a dose corresponding to the BSA (40 mg with a BSA <1.25 m^2^, 50 mg with a BSA of 1.25–1.50 m^2^ and 60 mg with a BSA >1.50 m^2^) twice daily after meals for 28 consecutive days, followed by a 14-day rest. Four courses were administered. The FU plus L-OHP regimens were as follows. FOLFOX regimen (n = 99): patients received a 2-h infusion of 85 mg/m^2^ of L-OHP with the LV5FU2 standard regimen given simultaneously with leucovorin. Twelve courses were administered. XELOX regimen (n = 3): patients received capecitabine (2000 mg/m^2^, biweekly) plus L-OHP (130 mg/m^2^, day 1). Eight courses were administered. L-OHP was discontinued in cases of persistent painful paresthesia or functional impairment.

Neoadjuvant chemoradiotherapy with S-1 (80 mg with a BSA <1.25 m^2^, 100mg with a BSA of 1.25–1.50 m^2^ and 120 mg with a BSA >1.50 m^2^ on days 1–5, 8–12, 22–26, and 29–33) and irradiation (total 45 Gy/25 fr, 1.8 Gy/day, on days 1–5, 8–12, 15–19, 22–26, and 29–33) or oral doxifluridine (900 mg/day) from days 1 to 35, and pelvic radiation of 45 Gy over 5 weeks were performed for lower rectal cancer.

### Follow-up

The standard follow-up methods in our institute consisted of computed tomography (CT) every 3 months for patients with stage IIIb CRC, 6 months for patients with stage III CRC classified according to the Japanese Society for the Cancer of the Colon and Rectum guidelines during the first 3 years after surgery; during the subsequent 2 years CT was carried out every 6 months, then annually. Evaluations were conducted independently of the type of chemotherapy and did not vary with time. Patient follow-up was defined as the time between surgery and the last hospital contact or disease recurrence.

### Statistical analysis

OS was defined as the time from the beginning of adjuvant chemotherapy until death from any cause; it was censored on the last day that the patient was alive. DFS was defined as the time from the surgery until objective tumor recurrence or death, whichever occurred first. OS and DFS were evaluated using the Kaplan–Meier method and compared using the log-rank test. Prognostic factors included: age (<65 or ≥65 years and <70 or ≥70 years); gender (male or female); family history (yes or no); pathology (well, mod or por, sig, muc); CEA (<5 or ≥5); CA19-9 (<37 or ≥37); lymph vascular invasion (yes or no); venous invasion (yes or no); region of colorectal cancer (left or right); bowel obstruction or leakage (yes or no); T-stage (T4 or others); N-stage (N1 or N2); collection of lymph nodes after surgery (<12 or ≥12); adjuvant chemotherapy (FU or addition of L-OHP); and <56 days until the start of adjuvant chemotherapy (yes or no). All reported *P*-values were the result of two-sided tests, with a *P* value < 0.05 considered as being statistically significant. Prognostic factors with *P* < 0.2 in the univariate analysis were included in the multivariate analysis. All statistical analyses were performed using EZR (Saitama Medical Center, Jichi Medical University, Tochigi, Japan), which is a graphical user interface for R (The R Foundation for Statistical Computing, Vienna, Austria).

## Results

### Patient characteristics

Patient characteristics are detailed in Table 1. Baseline characteristics were well balanced between the two groups, except for the frequency of usage of L-OHP. The data cutoff date was 31 September 2014. The median follow-up time was 92.3 months (range:1–97). A total of 509 patients were treated with FU based therapy with or without L-OHP. Patients received adjuvant chemotherapy over a 6-month period involving FU (n = 407) or FU plus L-OHP (n = 102). Completion rate of adjuvant chemotherapy was 76.7% (390/509).

**Table 1:** Patient characteristics.

	ITT population (n = 509)		*P* value
	2005–2007	2008–2010	
	(n = 189)	(n = 320)	
Sex, number (%)			1
Male	101 (53.4)	172 (53.7)	
Female	88 (46.6)	148 (46.3)	
Age (years)			
Median	61.5 (38–75)	55 (16–74)	
<65, number (%)	107 (56.6)	179 (55.9)	0.85
≥65, number (%)	81 (43.3)	141 (44.1)	
<70, number (%)	151 (79.9)	252 (78.8)	
≥70, number (%)	38 (20.1)	68 (21.2)	
Family history, n (%)			0.85
yes	99 (52.3)	171 (53.4)	
no	90 (47.6)	149 (46.6)	
Location of primary tumor, n (%)			0.44
Right	46 (24.3)	68 (21.2)	
Left	143 (75.7)	252 (78.8)	
Pathology, n (%)			0.51
well, mod	175 (92.6)	290 (90.6)	
por, sig, muc	14 (7.4)	30 (9.4)	
CEA level, n (%)			0.36
<5	137 (72.4)	219 (68.4)	
≥5	52 (27.6)	101 (31.6)	
CA19-9 level, n (%)			0.89
<37	162 (85.7)	278 (86.8)	
≥37	27 (14.3)	42 (13.2)	
Lymph vascular invasion, n (%)			0.67
yes	181 (95.7)	303 (94.6)	
no	8 (4.3)	17 (5.4)	
Collected lymph nodes after surgery, n (%)			0.63
<12	6 (3.2)	14 (4.3)	
≥12	183 (96.8)	306 (95.7)	
Bowel obstruction or leakage, n (%)			0.76
yes	5 (2.6)	7 (2.2)	
no	183 (97.4)	313 (97.8)	
Pathological stage, n (%)			0.42
IIIa (N1)	135 (71.4)	217 (67.8)	
IIIb (N2)	54 (28.6)	103 (32.2)	
UICC TNM stage			0.4
IIIA	46 (24.3)	64 (20)	
IIIB	100 (52.9)	171 (53.4)	
IIIC	43 (22.7)	85 (26.5)	
T-stage, n (%)			0.19
T1-3	151 (79.9)	239 (74.7)	
T4	38 (20.1)	81 (25.3)	
Adjuvant chemotherapy, n (%)			<0.05
FU	180 (95.2)	227 (71)	
FU plus L-OHP	9 (4.8)	93 (29)	
<56 days until start of adjuvant chemotherapy, n (%)			0.63
yes	157 (83)	259 (80.9)	
no	32 (17)	61 (19.1)	

Abbreviations: ITT, intention to treat; FU, 5- fluoropyrimidine; L-OHP, oxaliplatin; CEA, carcinoembryonic antigen; CA19-9, carbohydrate antigen 19–9

### Transition of adjuvant chemotherapy

**Fig 1:**
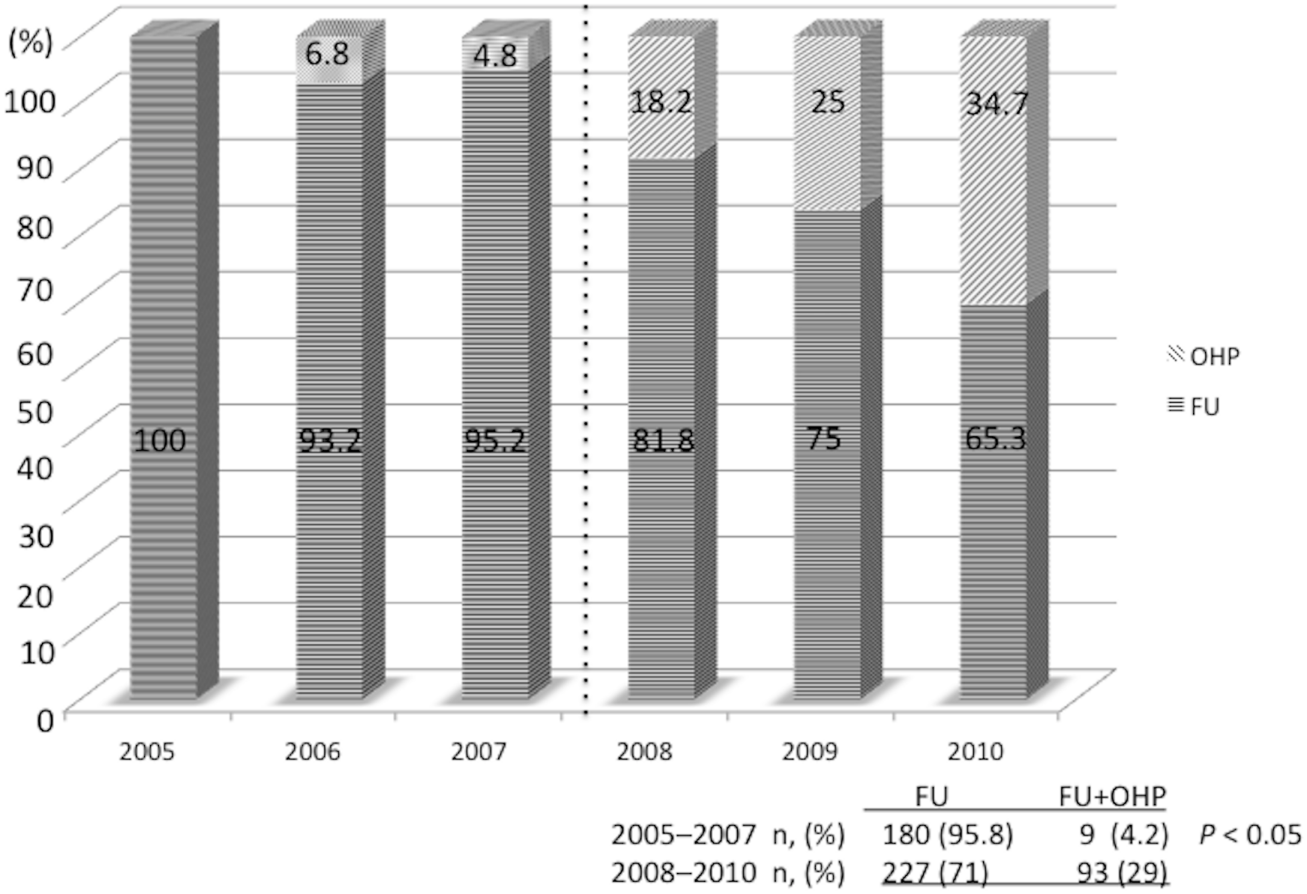
Transition in the frequency of administration of fluoropyrimidines alone or with oxaliplatin as adjuvant chemotherapy during the periods 2005–2007 and 2008–2010.

The frequency of use of L-OHP was increased after 2008, and a comparison of patient outcomes was carried out before and after this date. There was a significant difference between the two groups regarding both the ratio and the number of patients who were treated with L-OHP (2005–2007, 9/189 [4.2%]; 2008–2010, 93/320 [29%]; odds ratio, 0.12; 95%CI, 0.05–0.25; *P* < 0.05) (Fig 1) The ratio and the number of patients who were treated with L-OHP in both the JSCCR 8^th^ and UICC TNM stages were as follows: JSCCR 8^th^ stage IIIa 2005–2007, 5/135 (3.7%); 2008–2010, 25/217 (11.5%). stage IIIb 2005–2007, 4/54 (7.4%); 2008–2010, 68/103 (66%). UICC TNM stage IIIA 2005–2007, 0/46 (0%); 2008–2010, 4/64 (6.2%). stage IIIB 2005–2007, 6/100 (6%); 2008–2010, 31/171 (18.1%). stage IIIC 2005–2007, 3/43 (6.9%); 2008–2010, 58/85 (68.2%).

### DFS

**Fig 2:**
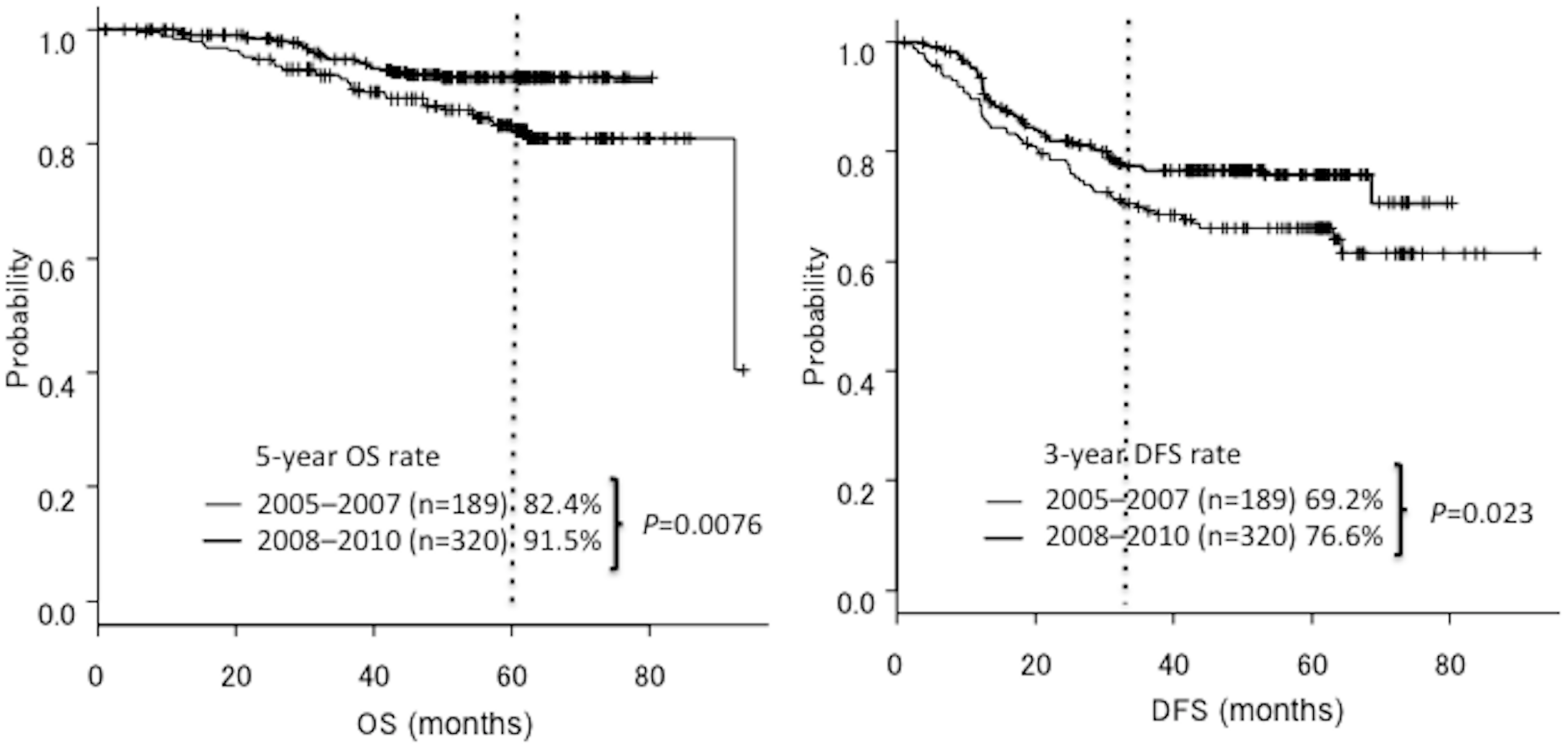
Overall survival and disease-free survival curves for the periods 2005–2007. (n = 189) and 2008–2010 (n = 320).

**Fig 3:**
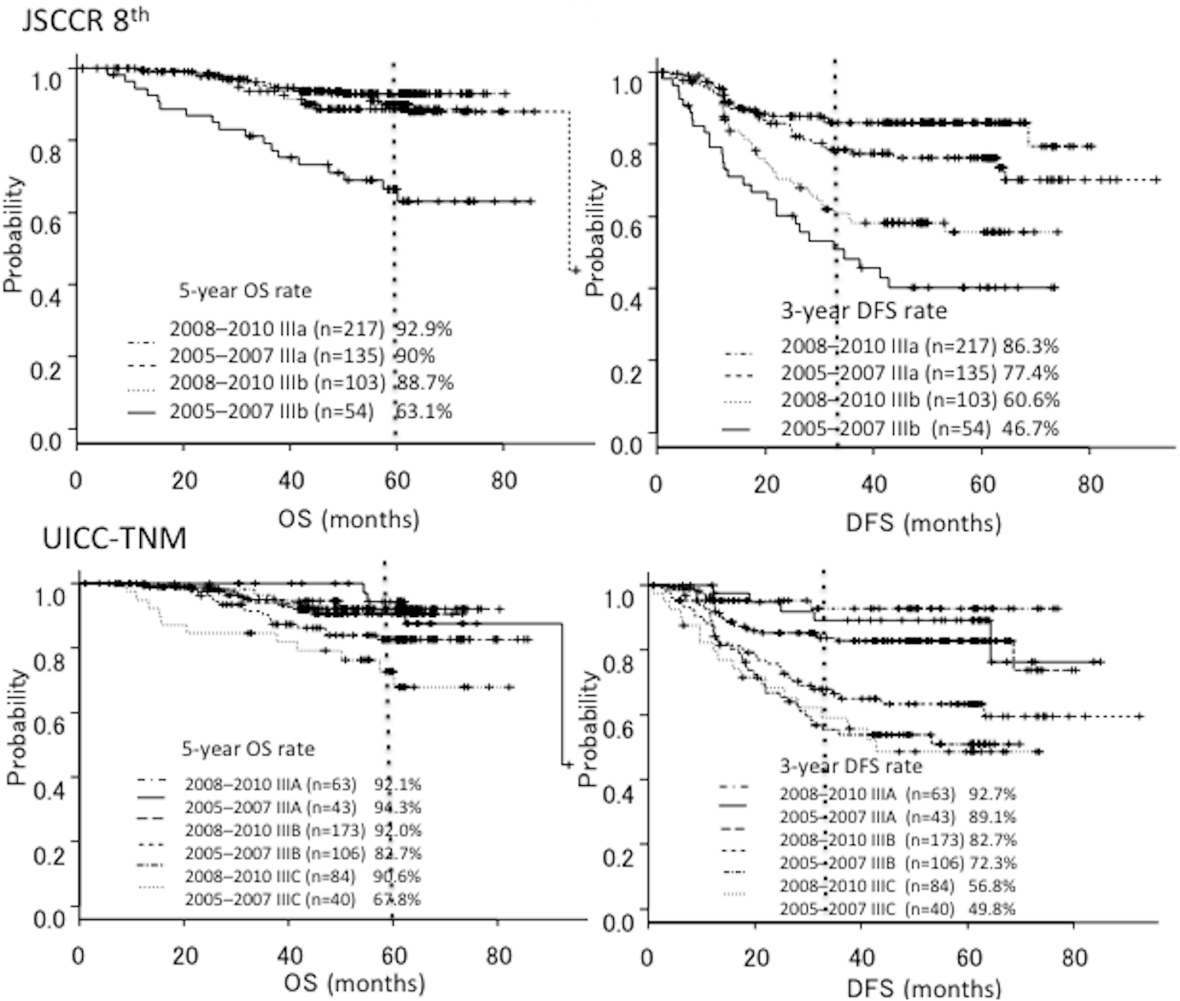
Overall survival and disease-free survival curves for the periods 2005–2007. (stage IIIa, n = 135; stage IIIb, n = 54), (stage IIIA, n = 43; stage IIIB, n = 106; stage IIIC, n = 40) and 2008–2010 (stage IIIa, n = 217; stage IIIb, n = 103), (stage IIIA, n = 63; stage IIIB, n = 173; stage IIIC, n = 84) in accordance with the Japanese Society for Cancer of the Colon and Rectum guidelines 2014 and the UICC-TNM classification 7th edition.

The 3-year DFS rate for all patients was 74.5% (95%CI, 0.7–0.78). The 3-year DFS rates were 76.6% (95%CI, 0.72–0.82) for the group treated from 2008 to 2010 and 69.2% (95%CI, 0.61–0.75) for the group treated from 2005 to 2007 (HR = 0.66; 95%CI, 0.46–0.94; *P* = 0.023) (Fig 2). In comparing patients with stage IIIa and stage IIIb CRC in each group, the 3-year DFS rates were 86.3% and 60.6%, respectively for the group treated from 2008 to 2010, and 77.4% and 46.7%, respectively for the group treated from 2005 to 2007 (stage IIIa, HR = 0.58; 95%CI, 0.34–0.98; *P* = 0.01, and stage IIIb, HR = 0.58; 95%CI, 0.35–0.96; *P* = 0.04) (Fig 3). In comparing UICC stage IIIA, IIIB and IIIC in each group, the 3-year DFS rates were 92.7%, 82.7% and 56.8%, respectively for the group treated from 2008 to 2010, and 89.1%, 72.3% and 49.8%, respectively for the group treated from 2005 to 2007 (stage IIIA, HR = 0.66; 95%CI, 0.46–0.94; *P* = 0.02, stage IIIB, HR = 0.44; 95%CI, 0.26–0.72; *P* = 0.001, and stage IIIC, HR = 0.99; 95%CI, 0.54–1.8; *P* = 0.97) (Fig 3). Table 2 showed the 3-year DFS of each T and N stage.

**Table 2:** 3 years DFS rate of stage III colorectal cancer.

		N1		N2	
		N1a	N1b	N2a	N2b
		(LNM:1)	(LNM:2–3)	(LNM:4–6)	(LMN:7-)
T1(SM)		90 (27/30)	100 (12/12)	100 (1/1)	-
		(77.7:95.2)	(100:100)	(-:100)	
T2(MP)		93.5 (58/62)	84.6 (22/26)	66.6 (4/6)	-
		(95:90.9)	(75:92.8)	(50:75)	
T3(SS/A)		81.5 (75/92)	86.1 (87/101)	57.1 (36/63)	42.1 (8/19)
		(73.5:86.2)	(76.9:91.9)	(45.8:64.1)	(50:36.3)
T4	T4a	65.2 (15/23)	68.7 (22/32)	58.6 (17/29)	30.7 (4/13)
	T4b	100 (5/5)	66.6 (8/12)	100 (2/2)	33.3 (1/3)
		(100:100)	(100:55.5)	(-:100)	(-:33.3)
JSCCR 8^th^		IIIa		IIIb	
UICC		IIIA	IIIB	IIIC	

### OS

The 5-year OS rate for all patients was 87.5% (95%CI, 0.83–0.90). The 5-year OS rates were 91.5% (95%CI, 0.87–0.94) for the group treated from 2008–2010 and 82.4% (95%CI, 0.75–0.87) for the group treated from 2005 to 2007 (HR = 0.48; 95%CI, 0.28–0.83; *P* = 0.0076) (Fig 2). In comparing patients with stage IIIa and stage IIIb CRC, the 5-year OS rates were 92.9% and 88.7%, respectively for the group treated from 2008 to 2010, and 90% and 63.1%, respectively for the group treated from 2005 to 2007 (stage IIIa, HR = 1.07; 95%CI, 0.43–2.60; *P* = 0.87, and stage IIIb, HR = 0.27; 95%CI, 0.11–0.65; *P* = 0.003) (Fig 3). In comparing patients with UICC stage IIIA, stage IIIB and stage IIIC CRC, the 5-year OS rates were 92.1%, 92% and 90.6%, respectively for the group treated from 2008 to 2010, and 94.3%, 82.7% and 67.8%, respectively for the group treated from 2005 to 2007 (stage IIIA, HR = 1.2; 95%CI, 0.28–5.6; *P* = 0.76; stage IIIB, HR = 0.47; 95%CI, 0.22–0.98; *P* = 0.04; and stage IIIC, HR = 0.37; 95%CI, 0.13–1.0; *P* = 0.06) (Fig 3).

### Univariate and multivariate analysis (Tables 3 and 4)

**Table 3:** Univariate and multivariate analysis for overall survival.

OS	Univariate analysis			p.value	Multivariate analysis			p.value
	HR	Lower 95%CI	Upper 95%CI		HR	Lower 95%CI	Upper 95%CI	
Age (<65 or ≥65 years)	1.35	0.74	2.4	0.31				
Sex (male or female)	1.08	0.59	1.97	0.79				
Family history (yes or no)	1	0.55	1.82	0.99				
Pathology (por, sig, muc or well, mod)	0.22	0.11	0.45	<0.001	0.28	0.13	0.59	<0.001
CEA (<5 or ≥5)	0.6	0.33	1.1	0.1	0.65	0.34	1.2	0.2
CA19-9 (<37 or ≥37)	0.4	0.21	0.79	0.008	0.6	0.29	1.23	0.16
Lymph vascular invasion (yes or no)	6.2	0.85	45.1	0.07	4	0.54	29.6	0.17
Venous invasion (yes or no)	1.56	0.65	3.6	0.31				
Region of colorectal cancer (left or right)	1.08	0.53	2.1	0.82				
Bowel obstruction or leakage (yes or no)	0.92	0.12	6.6	0.93				
T-stage (T4 or others)	2	1.1	3.8	0.02	1.5	0.79	2.8	0.2
N-stage (N1 or N2)	2.5	1.39	4.6	0.002	2	1.1	3.8	0.02
Collected lymph nodes after surgery (<12 or ≥12)	1.1	0.28	4.8	0.83				
Adjuvant chemotherapy (5-FU or addition of L-OHP)	0.88	0.40	1.91	0.75				
Within 56 days until the start of adjuvant chemotherapy (yes or no)	0.52	0.27	1.02	0.06	0.68	0.34	1.3	0.29

**Table 4:** Univariate and multivariate analysis for disease-free survival.

DFS	Univariate analysis			p.value	Multivariate analysis			p.value
	HR	Lower 95%CI	Upper 95%CI		HR	Lower 95%CI	Upper 95%CI	
Age (<65 or ≥65 years)	0.84	0.59	1.22	0.37				
Sex (male or female)	1.24	0.86	1.78	0.23				
Family history (yes or no)	0.8	0.56	1.1	0.22				
Pathology (por, sig, muc or well, mod)	0.32	0.2	0.5	<0.001	0.47	0.29	0.75	0.0001
CEA (<5 or ≥5)	0.65	0.45	0.93	0.02	0.79	0.53	1.17	0.24
CA19-9 (<37 or ≥37)	0.54	0.35	0.84	0.007	0.78	0.48	1.25	0.31
Lymph vascular invasion (yes or no)	1.9	1	3.9	0.04	1.23	0.61	2.4	0.56
Venous invasion (yes or no)	2.62	1.4	4.7	0.001	2.06	1.12	3.77	0.01
Region of colorectal cancer (left or right)	1.49	1	2.2	0.04	1.21	0.79	1.86	0.37
Bowel obstruction or leakage (yes or no)	1.06	0.33	3.3	0.91				
T-stage (T4 or others)	2	1.4	2.9	0.0001	1.5	1.02	2.2	0.03
N-stage (N1 or N2)	3.32	2.3	4.75	<0.001	2.5	1.7	3.7	<0.001
Collected lymph nodes after surgery (<12 or ≥12)	1.77	0.78	4	0.17	2	0.91	4.8	0.08
Adjuvant chemotherapy (5-FU or addition of L-OHP)	0.78	0.21	2.3	0.29				
Within 56 days until the start of adjuvant chemotherapy (yes or no)	0.95	0.60	1.5	0.84				

In the univariate analysis, pathology, CEA, CA19-9, lymph vascular invasion, venous invasion, region of colorectal cancer, T-stage, and N-stage were predictive factors for DFS and pathology, CA19-9, T-stage, and N-stage were predictive factor of OS. In the multivariate analysis, pathology, venous invasion, T-stage, and N-stage were predictive factors of DFS and T-stage and N-stage were predictive factors of OS.

## Discussion

This is the first study to analyze the transition of adjuvant chemotherapy for stage III CRC in a Japanese clinical setting. It was found that the frequency of usage of L-OHP as adjuvant chemotherapy is increasing annually. Furthermore, both the OS and DFS times reported in the period 2008–2010 were longer than in the period 2005–2007. In multivariate analysis, the OS was predicted by means of N stage and pathology, and the DFS was predicted by means of N stage, T stage, pathology and venous invasion.

In the present study, the frequency of use of L-OHP was increased after 2008, and comparison of patient outcomes was carried out before and after this date. During these periods, surgical procedure was changed from open surgery to laparoscopic surgery dramatically, and the palliative chemotherapy and neoadjuvant chemoradiotherapy had been improved. As already mentioned, it is controversial as to whether L-OHP should be used as adjuvant chemotherapy for patients with stage III CRC in Japan. Yamada et al. suggested that while L-OHP is an effective agent, its expected benefit might not be the same in all patients. [14] Similarly, de Gramont et al. indicated that stage III CRC consists of subgroups of patients with various prognoses, and the expected benefits of L-OHP could vary according to subgroup stage. [20] In our study, although the frequency of L-OHP was increasing annually, it was no influence for clinical outcomes in this study (OS: HR 0.88, 95%CI 0.4–1.91, p = 0.75, DFS: HR 0.78, 95%CI 0.21–2.3, p = 0.29). However, HR of DFS in MOSAIC trial, NSABP C-07 trial and NO16968 trial were 0.76 (95%CI 0.62–0.92), 0.80 (95%CI 0.69–0.93) and 0.80 (95%CI 0.69–0.93) similar in this study. Although above other factors that changed during these period might be also influenced for clinical outcomes, as this study was small sample size about adjuvant chemotherapy, it will be necessary to reevaluate additional effect of L-OHP with more patients.

In the present study, multivariate analysis indicated that T4, N2, diffuse pathology and venous invasion were also influenced clinical outcomes in Japan as well as previous reports. T4 stage was the significant predictive factor for DFS and pathology was the significant predictive factor for both DFS and OS. Snaebjornsson et al. reported that pathological T4 stage, among many variables analyzed in patients with stages II and III colon cancer, is the most important indicator of poor prognosis. [21] They also reported that it had a significance equal to that of lymph node status. The 5-year cancer specific survival rate for patients with stage III colon cancer at pathological T1–T2 stage was 88% (95%CI, 71–100%); at pathological T4 stage, it was 30% (95%CI, 18–41%). T4 stage was a negative independent prognostic factor as was pathological lymph node metastasis, peritumoral lymphocytic infiltration, type and differentiation, tumor location and patient age in multivariate analysis. [21] Regarding diffuse type histology, several studies have reported that it was an independent predictive factor for poor prognosis in patients with stage III colon cancer. Ishihara et al. reported that poorly differentiated mucinous and signet ring cell adenocarcinomas (*n* = 213) were more advanced concerning the TNM stage, and showed worse disease-specific survival than well and moderately differentiated adenocarcinomas (*n* = 2,692), especially for those with a distal location. [22] Kim et al. reported that mucinous histology is an independent predictive factor of poor prognosis regarding DFS in patients with stage III colon cancer after adjuvant FOLFOX chemotherapy (HR = 1.82; 95%CI, 1.03–3.23; *P* = 0.0403). [23] Furthermore, Hugen et al. reported that signet ring cell patients had poor 5-year relative survival of 30.8% (95%CI, 28.1–33.6%) in the colon and 19.5% (95%CI, 14.7–24.8%) in the rectum as compared with 56.8% (95%CI, 56.4–57.1%) and 58.5% (95%CI, 57.9–59.1%) for advanced colon cancer. [24] We compared the treatment outcomes of patients with these factors before and after 2008. Patients with N2 or venous invasion had a significant difference in DFS between the two groups, suggesting that adjuvant therapy including L-OHP might be one of the factors for longer DFS (S1 Fig). Patients with undifferentiated pathology had no significant difference in DFS, however in OS, there was significant difference between the two groups as well as patients with N2 (S1 Fig). It was the possibility that palliative chemotherapy after relapse might have influenced survival. In fact, in this study, the use frequency of molecular targeted drugs after relapse was higher in the latter period (2005–2007 61.9% (26/42), 2008–2010 79.2%(42/53), p = 0.07). Patients with T4 had no significant difference in both DFS and OS between the two groups (S1 Fig). Therefore, it will be necessary and expected to perform more intensive perioperative treatment.

The current study had several limitations. Sample size was small for an adjuvant chemotherapy study. In addition, it did not involve a direct comparison between the FU and FU plus L-OHP groups as adjuvant chemotherapy. Because we tended to use FU plus L-OHP for patients with a poor prognosis, especially stage IIIb, it was difficult to compare two groups because the background was quite different (S1 Table). In addition, our study included patients with CRC involving the lower part of the rectum (Rb). Chemoradiotherapy and subsequent proctectomy is the standard treatment for patients with stage III CRC in the Rb. In the Japanese Society for Cancer of the Colon and Rectum Guidelines 2014 for the treatment of colorectal cancer, both colon cancer and rectal cancer are included, but are usually considered as colon cancer in the US and Europe. In our study, the proportion of patients with CRC in the Rb (relative to those with CRC in the upper part of the rectum) was comparable in the two treatment groups (2005–2007, 15.8% [30/189] and 2008–2010, 19.6% [63/320]; *P* = 0.34).

In conclusion, the use of L-OHP as adjuvant chemotherapy is increasing annually. OS and DFS during the period from 2008 to 2010 were significantly longer than during the period from 2005 to 2007. Although the frequency of L-OHP usage was increasing annually, it was no influence for clinical outcomes in this study. Therefore, it will be necessary to reevaluate additional effect of L-OHP with more patients and it is important that the results of ongoing ACTS-CC02 prospective clinical trial involving the comparison of FU and FU plus L-OHP as adjuvant chemotherapy for patients with stage IIIb CRC.

## Supporting information

S1 Fig Sub group analysis of overall survival and disease free survival in the group of 2008–2010 as compared with the group of 2005–2007. (JPG)

S1 Table Patients characteristics between FU monotherapy and FU plus L-OHP. (DOCX)

S2 Table Univariate and multivariate analysis for the periods 2005–2007 and 2008–2010. (DOCX)
