# Supplementary figures and images for: Change in clinical outcomes during the transition of adjuvant chemotherapy for stage III colorectal cancer
Source: PLoS One. 2017 May 31;12(5):e0176745. doi: 10.1371/journal.pone.0176745 (PMC5451009; doi:10.1371/journal.pone.0176745)

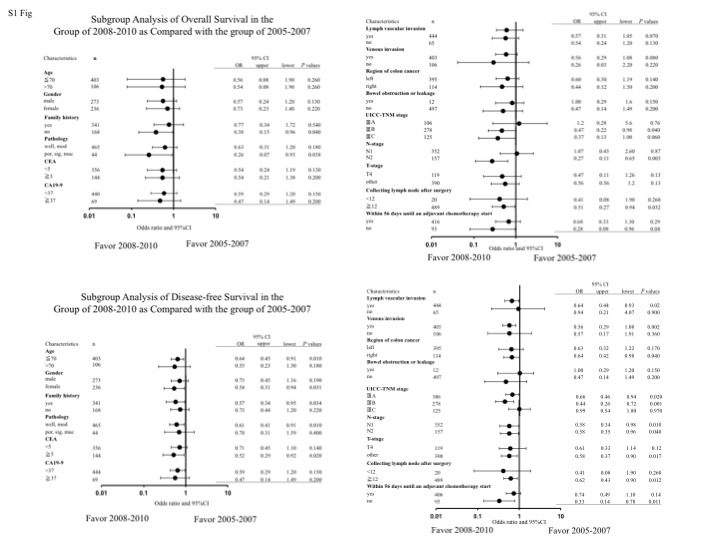

Supplement: S1 Fig — (JPG) [file pone.0176745.s001.jpg]
