# Supplementary material for: Change in clinical outcomes during the transition of adjuvant chemotherapy for stage III colorectal cancer
Source: PLoS One. 2017 May 31;12(5):e0176745. doi: 10.1371/journal.pone.0176745 (PMC5451009; doi:10.1371/journal.pone.0176745)
Supplement: S1 Table — (DOCX) [file pone.0176745.s002.docx]

| **S2 Table**  Patient characteristics | ITT population (n=509) | | | | |  |
| --- | --- | --- | --- | --- | --- | --- |
|  | FU | | | FU plus L-OHP | | |
|  | (n=407) | | | (n=102) | *P* value | |
| Sex, number (%) |  | | |  | 0.73 | |
| Male | 220 (54) | | | 53 (53.4) |  | |
| Female | 187 (46) | | | 49 (46.6) |  | |
| Age (years) |  | | |  |  | |
| Median | 55 (16–74) | | | 61.5 (38–75） |  | |
| <65, number (%) | 221 (54.3) | | | 66 (56.6) | 0.07 | |
| ≥65, number (%) | 186 (45.7) | | | 36 (43.3) |  | |
| <70, number (%) | 349 (85.7) | | | 90 (88.3) |  | |
| ≥70, number (%) | 58 (14.3) | | | 12 (11.7) |  | |
| Family history, n (%) |  | | |  | 0.73 | |
| yes | 214 (52.5) | | | 58 (52.3) |  | |
| no | 193 (47.5) | | | 46 (47.6) |  | |
| Location of primary tumor, n (%) |  | | |  | 0.18 | |
| Right | 86 (21.1) | | | 28 (24.3) |  | |
| Left | 321 (78.9) | | | 74 (75.7) |  | |
| Pathology, n (%) |  | | |  | <0.05 | |
| well, mod | 377 (92.6) | | | 88 (92.6) |  | |
| por, sig, muc | 30 (7.4) | | | 14 (7.4) |  | |
| CEA level, n (%) |  | | |  | <0.05 | |
| <5 | 296 (72.7) | | | 60 (58.8) |  | |
| ≥5 | 111 (27.3) | | | 42 (41.2) |  | |
| CA19-9 level, n (%) |  | | |  | <0.05 | |
| <37 | 361 (88.7) | | | 79 (77.4) |  | |
| ≥37 | 46 (11.3) | | | 23 (22.6) |  | |
| Lymph vascular invasion, n (%) |  | | |  | <0.05 | |
| yes | 348 (85) | | | 96 (94.1) |  | |
| no | 59 (15) | | | 6 (5.9) |  | |
| Venous invasion, n (%) |  | | |  | <0.05 | |
| yes | 307 (76.3) | | | 96 (94.1) |  | |
| no | 100 (23.7) | | | 6 (5.9) |  | |
| Collection of lymph nodes after surgery, n (%) |  | | |  | 0.77 | |
| <12 | 17 (4.1) | | | 3 (3) |  | |
| ≥12 | 390 (95.9) | | | 99 (97) |  | |
| Bowel obstruction or leakage, n (%) |  | | |  | 0.07 | |
| yes | 7 (1.7) | | | 5 (4.9) |  | |
| no | 400 (98.3) | | | 97 (95.1) |  | |
| Pathological stage, n (%) |  | | |  | <0.05 | |
| IIIa (N1) | 322 (79.1) | | | 30 (29.4) |  | |
| IIIb (N2) | 85 (20.9) | | | 72 (70.6) |  | |
| UICC stage |  | | |  | <0.05 | |
| IIIA | 106 (26.0) | | | 4 (3.9) |  | |
| IIIB | 234 (57.4) | | | 37(36.2) |  | |
| IIIC 67 (16.4) | | |  | 61 (59.8) |  | |
| T-stage, n (%) | |  | |  | <0.05 | |
| T1-3 329 (80.8) | |  | | 61 (59.8) |  | |
| T4 78 (19.2) | |  | | 41 (40.2) |  | |
| ˂56 days until start of adjuvant chemotherapy, n (%) | |  | |  | <0.05 | |
| yes 342 (85) | |  | | 74 (72.5) |  | |
| no 65 (15) | |  | | 28 (17.5) |  | |

Abbreviations: ITT, intention to treat; FU, 5-fluoropyrimidine; L-OHP, oxaliplatin; CEA, carcinoembryonic antigen; CA19-9, carbohydrate antigen 19-9
