# Supplementary material for: Change in clinical outcomes during the transition of adjuvant chemotherapy for stage III colorectal cancer
Source: PLoS One. 2017 May 31;12(5):e0176745. doi: 10.1371/journal.pone.0176745 (PMC5451009; doi:10.1371/journal.pone.0176745)
Supplement: S2 Table — (DOCX) [file pone.0176745.s003.docx]

**S3 Table**

Univariate and multivariate analysis for overall survival and disease-free survival in 2005-2007

| OS | Univariate analysis | | |  | Multivariate analysis | | |  |
| --- | --- | --- | --- | --- | --- | --- | --- | --- |
|  | HR | Lower 95%CI | Upper 95%CI | p.value | HR | Lower 95%CI | Upper 95%CI | p.value |
| Age (<65 or ≥65 years) | 0.98 | 0.43 | 2.25 | 0.97 |  |  |  |  |
| Sex (male or female) | 1.16 | 0.51 | 2.6 | 0.71 |  |  |  |  |
| Family history (yes or no) | 0.70 | 0.31 | 1.61 | 0.41 |  |  |  |  |
| Pathology (por, sig, muc or well, mod) | 0.13 | 0.05 | 0.33 | <0.001 | 0.28 | 0.13 | 0.59 | <0.001 |
| CEA (<5 or ≥5) | 0.58 | 0.25 | 1.3 | 0.21 |  |  |  |  |
| CA19-9 (<37 or ≥37) | 0.36 | 0.14 | 0.88 | 0.02 | 0.6 | 0.29 | 1.23 | 0.16 |
| Lymph vascular invasion (yes or no) | 6.0 | 0.89 | 37.2 | 0.09 | 4.3 | 0.67 | 28.2 | 0.13 |
| Venous invasion (yes or no) | 1.44 | 0.53 | 3.88 | 0.47 |  |  |  |  |
| Region of colorectal cancer (left or right) | 1.22 | 0.48 | 3.1 | 0.67 |  |  |  |  |
| Bowel obstruction or leakage (yes or no) | 0.90 | 0.10 | 7.2 | 0.89 |  |  |  |  |
| T-stage (T4 or others) | 2.3 | 0.98 | 5.4 | 0.053 | 1.5 | 0.79 | 2.8 | 0.2 |
| N-stage (N1 or N2) | 3.7 | 1.6 | 8.4 | 0.001 | 2 | 1.1 | 3.8 | 0.02 |
| Collected lymph nodes after surgery (<12 or ≥12) | 1.3 | 0.39 | 6.2 | 0.79 |  |  |  |  |
| Adjuvant chemotherapy (5-FU or addition of L-OHP) | 0.88 | 0.40 | 1.91 | 0.75 |  |  |  |  |
| Within 56 days until the start of adjuvant chemotherapy (yes or no) | 0.34 | 0.14 | 0.80 | 0.014 | 0.42 | 0.17 | 1.02 | 0.06 |

| DFS | Univariate analysis | | | |  | Multivariate analysis | | |  |
| --- | --- | --- | --- | --- | --- | --- | --- | --- | --- |
|  | | HR | Lower 95%CI | Upper 95%CI | p.value | HR | Lower 95%CI | Upper 95%CI | p.value |
| Age (<65 or ≥65 years) | | 0.84 | 0.59 | 1.22 | 0.37 |  |  |  |  |
| Sex (male or female) | | 1.16 | 0.51 | 2.6 | 0.71 |  |  |  |  |
| Family history (yes or no) | | 0.92 | 0.55 | 1.5 | 0.76 |  |  |  |  |
| Pathology ( por, sig, muc or well, mod) | | 0.32 | 0.2 | 0.5 | <0.001 | 0.46 | 0.22 | 0.93 | 0.03 |
| CEA (<5 or ≥5) | | 0.51 | 0.30 | 0.87 | 0.01 | 0.82 | 0.47 | 1.4 | 0.51 |
| CA19-9 (<37 or ≥37) | | 0.51 | 0.27 | 0.98 | 0.04 | 0.80 | 0.39 | 1.61 | 0.53 |
| Lymph vascular invasion (yes or no) | | 2.13 | 0.66 | 6.8 | 0.20 | 1.26 | 0.38 | 4.1 | 0.69 |
| Venous invasion (yes or no) | | 3.0 | 1.4 | 6.4 | 0.003 | 2.56 | 1.2 | 5.46 | 0.01 |
| Region of colorectal cancer (left or right) | | 1.54 | 0.87 | 2.7 | 0.13 | 1.33 | 0.71 | 2.4 | 0.42 |
| Bowel obstruction or leakage (yes or no) | | 1.06 | 0.33 | 3.3 | 0.91 |  |  |  |  |
| T-stage (T4 or others) | | 2.0 | 1.1 | 3.6 | 0.01 | 1.48 | 0.82 | 2.6 | 0.18 |
| N-stage (N1 or N2) | | 2.84 | 1.68 | 4.78 | <0.001 | 2.2 | 1.2 | 3.8 | 0.004 |
| Collected lymph nodes after surgery (<12 or ≥12) | | 1.3 | 0.33 | 5.5 | 0.66 |  |  |  |  |
| Adjuvant chemotherapy (5-FU or addition of L-OHP) | | 1.2 | 0.41 | 3.8 | 0.67 |  |  |  |  |
| Within 56 days until the start of adjuvant chemotherapy (yes or no) | | 0.61 | 0.32 | 1.1 | 0.13 | 0.60 | 0.30 | 1.1 | 0.12 |

Univariate and multivariate analysis for overall survival and disease-free survival in 2008-2010

| OS | Univariate analysis | | |  | Multivariate analysis | | |  |
| --- | --- | --- | --- | --- | --- | --- | --- | --- |
|  | HR | Lower 95%CI | Upper 95%CI | p.value | HR | Lower 95%CI | Upper 95%CI | p.value |
| Age (<65 or ≥65 years) | 1.02 | 0.42 | 2.4 | 0.95 |  |  |  |  |
| Sex (male or female) | 1.08 | 0.59 | 1.97 | 0.79 |  |  |  |  |
| Family history (yes or no) | 1.5 | 0.61 | 3.8 | 0.35 |  |  |  |  |
| Pathology ( por, sig, muc or well, mod) | 0.32 | 0.10 | 0.96 | 0.04 | 0.30 | 0.09 | 0.99 | 0.05 |
| CEA (<5 or ≥5) | 0.58 | 0.24 | 1.4 | 0.22 |  |  |  |  |
| CA19-9 (<37 or ≥37) | 0.48 | 0.17 | 0.34 | 0.16 | 0.51 | 0.17 | 1.48 | 0.21 |
| Lymph vascular invasion (yes or no) | 3.2 | 0.44 | 24.6 | 0.24 |  |  |  |  |
| Venous invasion (yes or no) | 3.1 | 0.42 | 23.6 | 0.26 |  |  |  |  |
| Region of colorectal cancer (left or right) | 0.92 | 0.30 | 2.7 | 0.88 |  |  |  |  |
| Bowel obstruction or leakage (yes or no) | 0.92 | 0.12 | 6.6 | 0.93 |  |  |  |  |
| T-stage (T4 or others) | 1.99 | 10.81 | 4.8 | 0.12 | 1.83 | 0.73 | 4.5 | 0.19 |
| N-stage (N1 or N2) | 1.7 | 0.72 | 4.3 | 0.2 | 1.19 | 0.45 | 3.1 | 0.71 |
| Collected lymph nodes after surgery (<12 or ≥12) | 2.4 | 0.57 | 10.6 | 0.22 |  |  |  |  |
| Adjuvant chemotherapy (5-FU or addition of L-OHP) | 1.21 | 0.38 | 3.8 | 0.75 |  |  |  |  |
| Within 30 days until the start of adjuvant chemotherapy (yes or no) | 0.87 | 0.29 | 2.6 | 0.73 |  |  |  |  |

| DFS | Univariate analysis | | | |  | Multivariate analysis | | |  |
| --- | --- | --- | --- | --- | --- | --- | --- | --- | --- |
|  | | HR | Lower 95%CI | Upper 95%CI | p.value | HR | Lower 95%CI | Upper 95%CI | p.value |
| Age (<65 or ≥65 years) | | 0.84 | 0.59 | 1.22 | 0.37 |  |  |  |  |
| Sex (male or female) | | 1.44 | 0.86 | 2.3 | 0.15 |  |  |  |  |
| Family history (yes or no) | | 0.73 | 0.44 | 1.2 | 0.22 |  |  |  |  |
| Pathology (por, sig, muc or well, mod) | | 0.29 | 0.16 | 0.53 | <0.001 | 0.42 | 0.22 | 0.81 | 0.01 |
| CEA (<5 or ≥5) | | 0.74 | 0.44 | 1.2 | 0.26 |  |  |  |  |
| CA19-9 (<37 or ≥37) | | 0.56 | 0.30 | 1.03 | 0.06 | 0.75 | 0.40 | 1.42 | 0.38 |
| Lymph vascular invasion (yes or no) | | 1.7 | 0.74 | 4.0 | 0.19 | 0.96 | 0.40 | 2.33 | 0.9 |
| Venous invasion (yes or no) | | 2.76 | 1.0 | 7.6 | 0.048 | 2.12 | 0.75 | 5.97 | 0.15 |
| Region of colorectal cancer (left or right) | | 1.4 | 0.85 | 2.5 | 0.16 | 0.96 | 0.53 | 1.74 | 0.9 |
| Bowel obstruction or leakage (yes or no) | | 1.06 | 0.33 | 3.3 | 0.91 |  |  |  |  |
| T-stage (T4 or others) | | 2.2 | 1.3 | 3.6 | 0.001 | 1.71 | 1.0 | 2.9 | 0.05 |
| N-stage (N1 or N2) | | 4.0 | 2.4 | 6.6 | <0.001 | 2.9 | 1.7 | 5.0 | <0.001 |
| Collected lymph nodes after surgery (<12 or ≥12) | | 1.7 | 0.62 | 4.7 | 0.30 |  |  |  |  |
| Adjuvant chemotherapy (5-FU or addition of L-OHP) | | 0.9 | 0.49 | 1.64 | 0.73 |  |  |  |  |
| Within 56 days until the start of adjuvant chemotherapy (yes or no) | | 1.1 | 0.68 | 1.9 | 0.59 |  |  |  |  |
